# Developmental Coordination Disorder and Executive Function in Children with ADHD

**DOI:** 10.1192/j.eurpsy.2025.1113

**Published:** 2025-08-26

**Authors:** L. R. R. Carreiro, S. M. Blascovi-Assis, A. G. Seabra, M. C. T. V. Teixeira, M. Á. Muñoz

**Affiliations:** 1 Universidade Presbiteriana Mackenzie, São Paulo, Brazil; 2 Universidad de Granada, Granada, Spain

## Abstract

**Introduction:**

Developmental Coordination Disorder (DCD) is a motor skills disorder characterized by delayed motor development. It affects approximately 6% of school-aged children, limiting their ability to perform everyday tasks. DCD is often associated with Attention Deficit/Hyperactivity Disorder (ADHD) and executive functions prejudices, making it essential to conduct more detailed investigations.

**Objectives:**

To explore potential correlations and trends between suspected DCD and behaviors related to inattention, hyperactivity, and impulsivity, as well as performance on tasks that involve attention, cognitive flexibility, and working memory.

**Methods:**

The study utilized data from a protocol for ADHD assessment conducted at Mackenzie Center for Research in Childhood and Adolescence in São Paulo, Brazil, with approval from the Ethics Committee. The protocol consists of neuropsychological, behavioral and psychiatric assessments. For this study, the following tests were considered: Rey Complex Figure Test and the Five-Digit Test (FDT) to assess cognitive flexibility and memory, Psychological Battery for Attention (BPA) to assess attention, Total index of the ADHD questionnaire based on DSM-5 criteria (total items of greatest severity), and DCD Questionnaire for DCD assessment. Twelve children aged between 6 and 15 years, who were referred for evaluation due to complaints of inattention and hyperactivity, participated in this study. Pearson correlation analyses were performed between the DCDQ and the other collected data.

**Results:**

The results revealed a significant positive correlation between the DCDQ and the Rey Complex Figure Test (r=0.840, p=0.009), suggesting that better motor performance is associated with improved planning and memory functions. There was also a marginally significant correlation between the DCDQ and the BPA divided attention test (r=0.646, p=0.083), which engages working memory abilities. A marginally significant negative correlation was found between the DCDQ and the FDT cognitive flexibility test (r=-0.637, p=0.065), indicating that higher DCDQ scores were associated with shorter times to complete the test. A negative correlation was also observed for the most severe items of the ADHD questionnaire related to hyperactivity and impulsivity. However, inattention complaints did not correlate with the DCDQ scores.

**Conclusions:**

Developmental coordination disorders should not be overlooked in studies and assessment protocols for children with suspected ADHD. Further investigations are needed to identify the domains and characteristics most closely associated with DCD. This will provide evidence to support the development of care programs and intervention strategies for these children.

**Disclosure of Interest:**

None Declared

